# Physiological Effects of a Garden Plant Smellscape from the Perspective of Perceptual Interaction

**DOI:** 10.3390/ijerph20065004

**Published:** 2023-03-12

**Authors:** Xinguo Zhang, Jiayu Guo, Xiaowan Zhang, Qixiang Zhang

**Affiliations:** 1 College of Landscape Architecture, Beijing Forestry University, Beijing 100083, China; zhangxinguo@nwafu.edu.cn; 2 College of Landscape Architecture and Art, Northwest Agriculture & Forestry University, Yangling, Xianyang 712100, China

**Keywords:** restorative landscape, garden plant, smellscape, olfactory–visual stimulus, physiological relaxation

## Abstract

The purpose of this study was to investigate the physiological recovery effects of olfactory, visual and olfactory–visual stimuli associated with garden plants. In a randomized controlled study design, ninety-five Chinese university students were randomly selected to be exposed to stimulus materials, namely the odor of Osmanthus fragrans and a corresponding panoramic image of a landscape featuring the plant. Physiological indexes were measured by the VISHEEW multiparameter biofeedback instrument and a NeuroSky EEG tester in a virtual simulation laboratory. The results showed the following: (1) In the olfactory stimulation group, from before to during exposure to the stimuli, the subjects’ diastolic blood pressure (DBP) (ΔDBP = 4.37 ± 1.69 mmHg, *p* < 0.05) and pulse pressure (PP) values increased (ΔPP = −4.56 ± 1.24 mmHg, *p* < 0.05), while their pulse (*p*) values decreased (ΔP = −2.34 ± 1.16 bmp, *p* < 0.05) significantly. When compared to the control group, only the amplitudes of α and β brainwaves increased significantly (Δα = 0.37 ± 2.09 µV, Δβ = 0.34 ± 1.01 µV, *p* < 0.05). (2) In the visual stimulation group, the amplitudes of skin conductance (SC) (ΔSC = 0.19 ± 0.01 µΩ, *p* < 0.05), α brainwaves (Δα = 6.2 ± 2.26 µV, *p* < 0.05) and β brainwaves (Δβ = 5.51 ± 1.7 µV, *p* < 0.05) all increased significantly relative to the control group. (3) In the olfactory–visual stimulus group, DBP (ΔDBP = 3.26 ± 0.45 mmHg, *p* < 0.05) values increased, and PP values decreased (ΔPP = −3.48 ± 0.33 bmp, *p* < 0.05) significantly from before to during exposure to the stimuli. The amplitudes of SC (ΔSC = 0.45 ± 0.34 µΩ, *p* < 0.05), α brainwaves (Δα = 2.28 ± 1.74 µV, *p* < 0.05) and β brainwaves (Δβ = 1.4 ± 0.52 µV, *p* < 0.05) all increased significantly relative to the control group. The results of this study show that the interaction of olfactory and visual stimuli associated with a garden plant odor landscape was able to relax and refresh the body to a certain extent, and this physiological health effect was greater with regards to the integrated response of the autonomic nervous system and central nervous system than the effect of only smelling or viewing the stimuli. In the planning and designing of plant smellscapes in garden green space, it should be ensured that plant odors and corresponding landscapes are present at the same time in order to ensure the best health effect.

## 1. Introduction

Today, due to the rapid development of urbanization and diversified and complex social pressures, people’s various health risks are aggravated [1]. In this post-COVID period, public health has become a serious concern globally. An increasing number of researchers have attempted to improve living environments to help people relieve pressure and relax their bodies and minds. Urban green space environments are closely related to human health. Garden plants, as an important part of urban green space, are closely related to relieving pressure and inducing relaxation. In recent years, scholars have increasingly paid attention to the restorative effect of garden plants on human health, including the effects of smell, visual landscapes, etc., which are related to plants in garden environments [2,3,4,5,6,7,8].

Researchers have found that plant scents stimulate nerves in the brain through the olfactory sulci, inducing the central nervous system and endocrine system to secrete hormones, thereby affecting various physiological reactions [9]. Tong et al. found that the living aroma of rosemary and lemon grass can regulate the nervous system of the human body and induce an antidepression effect [10]. The odor of *Abies holophylla* Maxim. has been found to have a positive effect on the autonomic nervous system (blood pressure, heart rate variability, etc.), to alleviate stress and to improve vascular function [11]. Torii found that the scents of sandalwood, bergamot, lemon, marjoram, chamomile and lavender essential oils are calming [2]. Lehrner et al. tested the effect of aroma on the mood of patients waiting for dental treatment, and their results showed that orange and lavender smells seemed to reduce patients’ anxiety and significantly improve their mood [3].

In addition, many studies have shown that viewing landscapes of green spaces can reduce stress and promote mental health [12]. A previous study found that the visual stimulation of forest images resulted in a decrease in the concentration of oxygen Hb in the prefrontal cortex [13]. Watching videos of bamboo forests with a high canopy density (0.83–0.85) may significantly decrease α waves, relaxing the human body [14]. Li X, adopting a biofeedback measurement and psychological test, found that under the visual stimulation of flower plants and leaf plants of different kinds and colors, the blood pressure (BP), heart rate (HR) and brainwaves of subjects showed positive physiological responses; their anxiety, anger and fatigue were significantly reduced; and their vitality levels increased [15].

Previous studies have shown that the stimulation of plant smells or plant landscapes can affect the health of humans. Three kinds of indicators are used to measure human physiological health, namely autonomic nervous system (ANS) indicators, central nervous system (CNS) indicators and biological indicators reflecting the stress response. The autonomic nervous system is also known as the visceral or involuntary nervous system, and its function is not under conscious, voluntary control. The ANS is related to vital functions such as respiration, heart rate and blood pressure and plays an important role in dynamically controlling the body’s response to internal and external stimuli and in subtly regulating biological homeostasis [16]. The autonomic nervous system is mainly composed of the sympathetic nervous system (SNS) and the parasympathetic nervous system (PNS). In general, the SNS is activated by excitement and nervousness, and it is often accompanied by an increase in heart rate and blood pressure. The PNS, activated by relaxation, plays a dominant role in states of quiet rest and is associated with reduced heart rate and blood pressure [17].

The central nervous system, composed of the spinal cord and the brain, plays an important role in the pattern of human behavior. People’s hearing, tasting, smelling, feeling and seeing are all controlled by the central nervous system [18,19]. Indexes used to study the central nervous system generally include brainwave measurement (which can be recorded by an electroencephalogram, biofeedback instrument, brainwave instrument, etc.) [6,20], cerebral blood flow dynamics (judged by changes in hemoglobin concentration) [21], brain activation regions (judged by functional MRI and fMRI imaging of brain regions or near-infrared optical brain imaging systems) [22,23,24,25] and electromyography (EMG) [26]. In terms of the biological indicators of the stress response, currently, common indicators used in the study of the relationship between human and the natural environment include salivary alpha-amylase activity [27], salivary cortisol concentration, salivary immunoglobulin (IgA) concentration [21], etc.

‘It penetrates people’s hearts and spleens’ is a Chinese idiom used to describe the fragrance of plants as comforting [28]. The ‘heart’ and ‘spleen’ mentioned here are not the heart and spleen in the modern medical sense. In the theory of traditional Chinese medicine, it is believed that the ‘heart’ is closely related to mental thinking activities [29], which can be regarded as the function of the brain, namely the central nervous system in modern medicine. According to the theory of the ‘spleen main muscle’ in traditional Chinese medicine, the ‘spleen’ corresponds to the function of the autonomic nervous system in modern medicine. In this sense, interpreting the health benefits of odors from the central nervous system and autonomic nervous system dimensions has a long history in China. The traditional medical system of Western Europe also has a long history of research on the effects of plant components on the human central nervous system and autonomic nervous system [30,31]. At present, in the study of the physiological effects of smell or plant odors on the human body, a lot of attention has been paid to the central nervous system and the autonomic nervous system [11,31,32,33,34]. However, in the field of garden plant smellscapes, studies that focus on these systems are still scarce.

In addition, most previous studies on the restorative function of garden plants only focused on the olfactory or visual level, in other words, on a single dimension. As we know, people’s activities in urban green space involve comprehensive experiences of vision, smell and other senses [4,7,8,12,15,33]. In order to further study the relationship between green space and human health, it is necessary to move from studying only a single sensory dimension to analyzing the impact of multidimensional sensory interactions on the human body, such as olfactory and visual interaction.

Therefore, the aim of this study was to move past the single sensory dimension approach by adopting ‘multidimensional perception’ as its research core. We used the smell of a common Chinese garden plant (Osmanthus fragrans) and a panoramic image of the corresponding landscape (a garden landscape photograph with Osmanthus trees as the main content taken by a panoramic camera) as the stimulus materials and used a group of college students with normal olfactory and visual abilities to compare and analyze the effects of olfactory, visual and olfactory–visual stimuli on various physiological indexes. Our aim was to expound the relationship between garden plant smellscapes and human health in order to provide a theoretical basis for the objective evaluation of the effect of the smell landscapes of garden plants on human health and to guide the scientific construction of garden plant smell studies. The following specific questions were put forward: (1) Are people’s autonomic nervous systems or central nervous systems affected by their smelling an Osmanthus, viewing a landscape of an Osmanthus or smelling the plant’s fragrance while viewing the landscape? (2) If so, are there any differences among the above three cases? (3) If there are differences, are they manifested in the autonomic or central nervous system? What are the differences between the two nervous systems?

## 2. Materials and Methods

### 2.1. Subjects

This study was conducted among university students (undergraduate and graduate students) aged 18–26 years old; all subjects were recruited through the campus network and chose to participate in the experiment voluntarily and knowingly. After a preliminary interview, patients with rhinitis, cold and olfactory disorders were screened out. Subjects were required to have adequate sleep the day before the test, to not smoke or drink alcohol and to not use perfume or strong fragrance cosmetics on the day of the test. For some myopic subjects, after they were asked about their degree of myopia before the test, the focus of their virtual reality (VR) glasses was adjusted to ensure that the scene seen through their glasses was clear. In addition, subjects with color blindness or color weakness were excluded. The test for color blindness or weakness was conducted using the fifth edition of the Color Blindness Test Map [35]; this was carried out in the experimental preparation stage. If the subjects reported color blindness or color weakness, they were withdrawn from participating in the experiment. Finally, 95 students (24 men and 71 women) were selected to participate in the experiment. In order to ensure the effect of the experiment and avoid a practice effect caused by repeated participation of the subjects [36], the subjects in each group did not overlap.

### 2.2. Experimental Design

**Figure 1:**
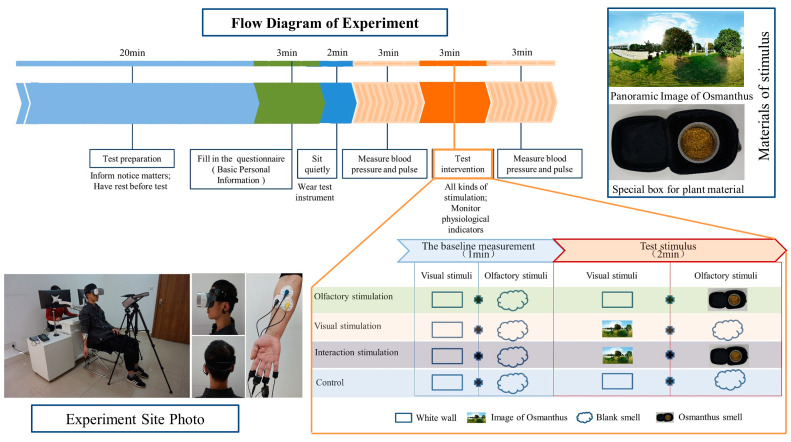
Experimental procedure.

All the data collected in this study were measured in the Virtual Simulation Room of the Landscape Architecture and Art College at Northwest A&F University, and all the participants were exposed to the same environmental conditions. The experiment was carried out in a room with white walls and ceilings, and the interior space was 4 m (long) × 3 m (wide) × 3.1 m (high). In order to prevent the mood of the subjects from being affected by weather and light, the indoor environment was kept the same: ambient light was 300 lx, ambient sound was not higher than 45 ± 5 dB, temperature was 25 ± 2 °C and relative humidity was 55 ± 5%. The physiological test instruments were placed behind the chair the subjects were sitting on, and on the opposite side of the chair was a white wall to avoid unnecessary visual influence (Figure 1).

A single-blind test was performed using a randomized and crossover design, and physiological reactions, including autonomic nervous system indicators and changes in the central nervous system, were measured. Participants were randomly assigned to different processing sequences (olfactory, visual, olfactory–visual stimulus sequences and the control group). In each group of experiments, subjects were randomly subjected to stimuli, and the flows of other steps were the same except for different stimulus types set in the intervention stage of the experiment, as shown in Figure 1. In the control group, the subjects sniffed blank scents and wore VR glasses to view the white lab wall. The average test time for each person was about 34 min. During each test interval, the door and window were opened for ventilation for 10 min to remove residual odor in the air. The research protocol was approved by the University Ethical Committee.

### 2.3. The Stimulus

The olfactory stimulus materials were the flowers of Osmanthus fragrans var. thunbergia, which is not only widely distributed but also one of the ten most famous flowers in China [37]. The flower is very commonly found in urban green space. In order to ensure the authenticity of the plant’s smell, plant materials were collected 0.5~1 h in advance on the test day and placed in a colorless and odorless cylindrical PE plastic airtight container with a volume of 600 mL. Flower volatiles are mixtures composed of various substances, and in nature, these are also mixed with air. The treatment method used in this study can truly reflect the influence of volatiles [38]. To prevent visual cues from influencing olfactory perception, a special box was added to the outside of the container (Figure 1), which was covered before the test and opened during the sniffing phase. The olfactory stimulation material device was fixed on a tripod with an adjustable height, and the horizontal distance between the device and the nose of the subject was 10 cm, while both were at the same height [31,39,40], which ensured that the sniffing radius of each subject was consistent. Olfactory stimulation odor concentration refers to the fragrance concentration of natural volatilization of Osmanthus fragrans in real environments of garden green space when there is no wind on sunny days. The data were measured by a COSMOS XP-329IIir (Osaka, Japan) portable odor sensor. The visual stimulus material was a panorama photo of Osmanthus trees, which was collected through the network and imported into virtual reality equipment (Figure 1). The olfactory–visual stimulus was a combination of visual and olfactory stimuli. The control group was exposed to white wall images in the laboratory under an odorless condition, which did not produce stimuli related to plant smells or a garden landscape. All visual stimuli were generated while the subject wore VR glasses.

### 2.4. Physiological Measurements

The selected physiological indicators were divided into autonomic nervous system (ANS)-related indicators (blood pressure, pulse pressure difference, pulse and skin conductance) and central nervous system (CNS)-related indicators (alpha brainwaves and beta brainwaves).

#### 2.4.1. Measurements of Indicators Related to the Autonomic Nervous System

After sitting for 20 min and taking a full rest, the subjects were fitted with equipment, and their blood pressure (BP) and pulse (P) were measured using an upper-arm electronic sphygmomanometer (OMRON, HEM-7211, Kyoto, Japan). Both BP and P were measured twice and averaged for analysis. After the intervention, BP and P were measured again. Pulse pressure difference (PP) data were calculated and recorded by the staff. As a basic physiological index of the human body, BP (including SBP and DBP) is often used to measure the relationship between garden plant smells and human health [11,30,31]. Excitation of sympathetic nerves is manifested by an increase in BP, and increased parasympathetic nerve activity is manifested by a decrease in BP [11]. PP is the difference between SBP and DBP, which, like blood pressure and pulse, can reflect the health of the human body [32]. Pulse is an important indicator of cardiovascular health. Generally speaking, when the human body is in a stressful state or environment, *p* values increase; otherwise, they fall [33].

Skin conductance (SC) was measured by a multiparameter biofeedback instrument (VISHEEW, Infiniti3000A, Nanjing, China), and these data were recorded by Bioneuro software(BioNeuro Infiniti v5.0), which is a physiological indicator that can reflect emotional stress and that is significantly affected by skin sweat secretion [34]. Before the experiment, the subjects were fitted with a biofeedback device, as shown in Figure 1. At baseline measurement and during the test stimulation, Bioneuro software was used to continuously record changes in the SC values of the subjects for 60 s and 120 s. Physiological psychologists measure the activity of sweat glands in order to study the related psychological activities [41]. While SC, which is controlled by the sympathetic nervous system, cannot function as the sole indicator of sympathetic activity [42], it is considered to be a reliable physiological measure of emotional arousal [43]. Under conditions of emotional excitement, tension, fear or anxiety, sweat gland secretion increases, and sweat on the skin surface increases, resulting in an increase in electrical conductivity and an increase in SC values. When a person’s mood is calm, their SC values decrease [44].

#### 2.4.2. Measurements of Indicators Related to the Central Nervous System

The brainwave types tested in this experiment were α waves and β waves, which were monitored by a brainwave device (made by NeuroSky (Silicon Valley, American), an American company; the device contains a TGAM brainwave chip) and numerically processed by the eSense TM algorithm. EEG signals are derived from the hyperpolarizing and depolarizing postsynaptic potentials in populations of pyramidal neurons, which exit the lower portion of the cerebral cortex [45]. An EEG can record these electrical signals from the scalp surrounding the human brain [46,47]. Data collected by an electroencephalogram (EEG) device when participants are faced with a stimulus are more objective and can better reflect participants’ true thoughts compared with data collected by traditional methods such as interviews or questionnaire surveys, and they are a kind of real-time physiological data [48]. Brainwaves are an internal scientific indicator of mood changes [49]; α waves are called ‘relaxing waves’ and ‘creative waves’ and are related to the active activity of the brain [50]; when the energy released by α waves is strong, this represents the brain being in a heightened state of learning and thinking. β waves are associated with concentration; when beta waves emit higher energy, they represent a positive increase in attention [51].

### 2.5. Statistical Analyses

Physiological indexes were analyzed and derived by the Bioneuro software platform and the eSense TM algorithm platform, and the data were processed by SPSS25.0 software. A paired-sample *t*-test was used to analyze the influence of the three intervention methods on the physiological indexes of the subjects before and during stimulation, and ANOVA and an LSD post test were used to analyze the differences in the variation in each groups’ physiological indexes. Microsoft Office PowerPoint and Photoshop CS6 were used for graphs. In order to standardize the data, when analyzing the physiological indicators of the subjects, the physiological indicators of blank smell and white wall stimulus state in the early stage of the experiment were taken as the baseline. The value of the change in the data from the baseline (Δ) was obtained according to the following calculation method: Δ = PSV − BSV (Δ: the value of change in the data from the baseline; PSV: change in index data during stimulus; BSV: baseline value).

## 3. Results

### 3.1. Changes in Autonomic Nervous System Indicator Data

In this study, a paired-sample *t*-test was used to analyze the influence of olfactory, visual and olfactory–visual stimulation methods on the physiological indexes of the subjects before and during stimulation. Table 1 shows that from before to during olfactory stimulation, the subjects’ systolic blood pressure (SBP) values did not change significantly (before, 108.06 ± 10.91 mmHg; during, 107.88 ± 11.14 mmHg; *p* > 0.05), but their diastolic blood pressure (DBP) values increased significantly (before, 62.88 ± 8.41 mmHg; during, 67.25 ± 10.10 mmHg; *p* < 0.05), and their pulse pressure difference (PP) values (before, 45.19 ± 8.29 mmHg; during, 40.63 ± 7.05 mmHg; *p* < 0.05) and pulse (P) values (before, 77.67 ± 12.56 bmp; during, 75.33 ± 11.4 bmp; *p* < 0.05) decreased significantly. During visual stimulation, the subjects exhibited no significant changes in their SBP, DBP, PP and *p* values. During olfactory–vision stimulation, significant increases in the subjects’ DBP (before, 61.04 ± 6.93 mmHg; during, 64.30 ± 6.48 mmHg; *p* < 0.05) and SC values (before, 2.95 ± 2.14 µΩ; during, 3.40 ± 2.48 µΩ; *p* < 0.05) were recorded, and there was a significant decrease in their PP values (before, 44.76 ± 4.84 mmHg; during, 40.92 ± 5.17 mmHg; *p* < 0.05), while their SBP (before, 105.80 ± 7.92 mmHg; during, 105.22 ± 7.45 mmHg; *p* > 0.05) and *p* values (before, 72.28 ± 9.95 bmp; during, 71.56 ± 10.19 bmp; *p* > 0.05) did not change significantly. As shown in Table 1, the subjects exhibited no significant changes in their SC values during olfactory (before, 2.94 ± 2.92 µΩ; during, 2.78 ± 2.68 µΩ; *p* > 0.05) and visual stimulation (before, 3.03 ± 2.17 µΩ; during, 3.22 ± 2.18 µΩ; *p* > 0.05), while these values increased significantly during olfactory–vision stimulation (before, 2.95 ± 2.14 µΩ; during, 3.40 ± 2.48 µΩ; *p* < 0.05).

**Figure 2:**
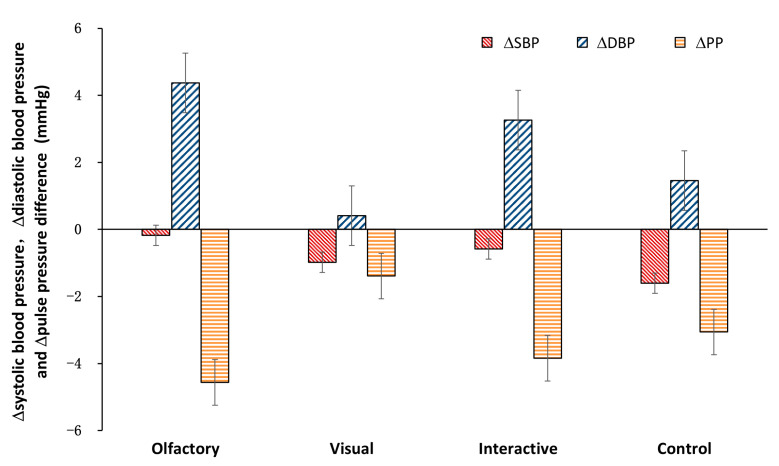
Effects of different stimulation types on human systolic blood pressure (SBP), diastolic blood pressure (DBP) and pulse pressure difference (PP).

**Figure 3:**
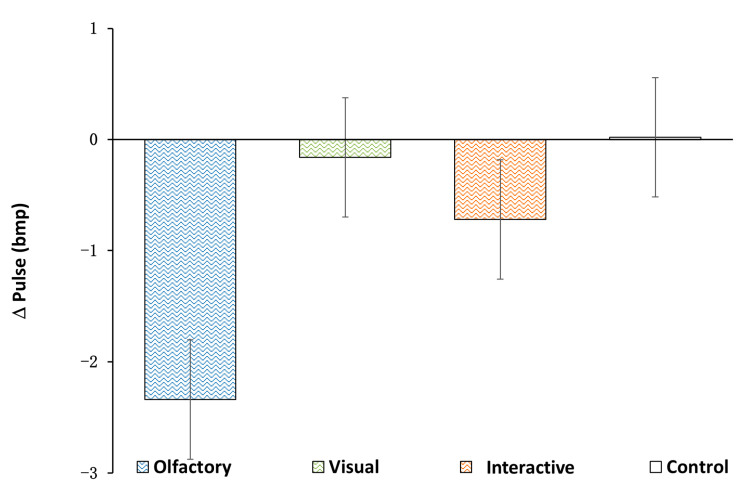
Effects of different stimulation types on human pulse (P).

**Figure 4:**
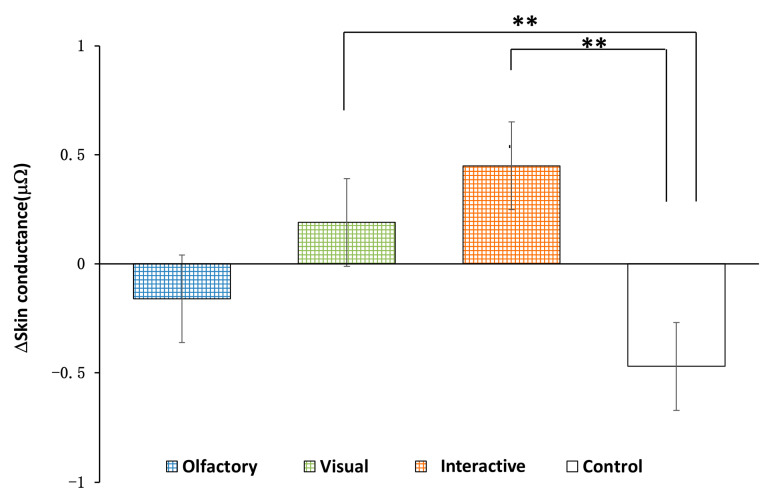
Effects of different stimulation types on human skin conductivity (SC). ** indicates that the difference between the two was extremely significant, *p* < 0.01.

The differences in the variation in each group’s physiological indexes were analyzed through ANOVA and an LSD post test. Among the three stimulation conditions, there were no significant differences relative to the mean values from the baseline in the blood pressure, pulse pressure difference and pulse values (ΔSBP: control, −1.60 ± 1.14 mmHg; olfactory, −0.19 ± 1.01 mmHg; visual, −0.98 ± 1.62 mmHg; olfactory–visual, −0.58 ± 1.00 mmHg; *p* > 0.05, Figure 2; ΔDBP: control, 1.46 ± 1.03 mmHg; olfactory, 4.38 ± 1.29 mmHg; visual, 0.41 ± 2.04 mmHg; olfactory–visual, 3.26 ± 1.17 mmHg; *p* > 0.05, Figure 2; ΔPP: control, −3.06 ± 1.06 mmHg; olfactory, −4.56 ± 1.30 mmHg; visual, −1.39 ± 2.41 mmHg; olfactory–visual, −3.84 ± 1.14 mmHg; *p* > 0.05, Figure 2; ΔP: control, 0.02 ± 1.01 bmp; olfactory, −2.33 ± 0.81 bmp; visual, −0.15 ± 0.95 bmp; olfactory–visual, −0.72 ± 0.76 bmp; *p* > 0.05, Figure 3). Furthermore, the average SC values relative to the baseline during the stimulus period were calculated for each participant; the mean values of these averages are shown in Figure 4. Both the visual stimulation and the olfactory–visual stimulation conditions showed significant differences relative to the control condition (ΔSC: control, −0.47 ± 0.01 µΩ visual, 0.19 ± 0.01 µΩ; olfactory–visual, 0.45 ± 0.34 µΩ; *p* < 0.001). No significant difference was observed for the olfactory stimulation condition.

### 3.2. Changes in Central Nervous System Indicator Data

As shown in Table 1, the subjects exhibited significant increases in both α-wave and β-wave values from before to during visual and olfactory–visual stimulation (α waves: visual, before, 13.90 ± 7.19 µV; during, 20.10 ± 9.45 µV, *p* < 0.001; olfactory–visual, before, 16.03 ± 5.03 µV; during, 18.31 ± 6.77 µV, *p* < 0.05; β waves: visual, before, 8.27 ± 5.09 µV; during, 13.78 ± 6.79 µV, *p* < 0.001; olfactory–visual, before, 10.12 ± 4.35 µV; during, 11.52 ± 4.87 µV, *p* < 0.05), but no significant changes in α-wave and β-wave values from before to during olfactory stimulation (α waves: before, 17.67 ± 6.64 µV; during, 18.04 ± 4.55 µV, *p* > 0.05; β waves: before, 11.49 ± 4.47 µV; during, 11.83 ± 5.48 µV, *p* > 0.05) were recorded.

**Figure 5:**
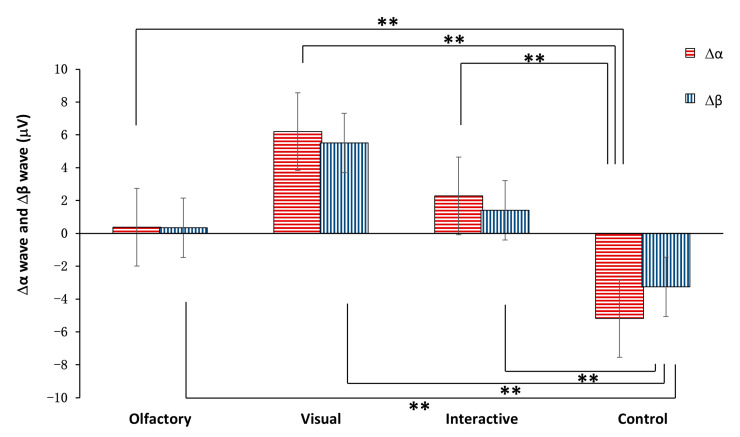
Effects of different stimulation types on the amplitudes of α and β brainwaves. ** indicates that the difference between the two was extremely significant, *p* < 0.01.

Figure 5 shows the overall α-wave and β-wave mean values relative to the baseline during the four stimulus conditions. All three stimulation conditions showed significant differences from the control condition (Δαwaves: control, −5.18 ± 6.64 µV; olfactory, 0.37 ± 2.09 µV, *p* < 0.05; visual, 6.20 ± 2.26 µV, *p* < 0.001; olfactory–visual, 2.28 ± 1.74 µV; *p* < 0.05, Figure 5; Δβ waves: control, −3.26 ± 3.5 µV; olfactory, 0.34 ± 1.01 µV, *p* < 0.05; visual, 5.51 ± 1.7 µV, *p* < 0.001; olfactory–visual, 1.40 ± 0.52 µV, *p* < 0.05, Figure 5).

### 3.3. Differences in Physiological Effects of Different Stimuli Related to Garden Plant Smellscape

Through one-way analysis of variance (ANOVA), we found that there were significant differences in three physiological indexes, i.e., SC (*p* = 0.000), α waves (*p* = 0.000) and β waves (*p* = 0.000), between the control group and each stimulus group.

There were three combinations that recorded significant differences in changes in skin conductivity (Table 2). In addition to the differences with regards to the control group, significant differences were found between the olfactory and olfactory–visual stimulus responses. There were four combinations that recorded significant differences in the changes in α brainwave amplitude. In addition to the differences with regards to the control group, significant differences were found between the olfactory and visual stimulus modes. In terms of the values of the changes in β brainwave amplitude, there were significant differences among the five combinations, and there were two combinations of different stimulus modes involved, namely the combination of the visual and olfactory stimulus modes and the combination of the visual and the olfactory–visual stimulus modes. 

## 4. Discussion

### 4.1. Effects of Olfactory, Visual and Olfactory–Visual Stimuli on the Autonomic Nervous System

Regarding the indicators selected in this study, olfactory stimulation had no significant effect on the autonomic nervous system, while both visual stimulation and olfactory–visual stimulation significantly affected the autonomic nervous system. The ANOVA of the variation in each ANS index revealed that except for the SC values, the variation in the BP, PP and *p* values was not significantly different from that of the control group. For the SC values, except for olfactory stimulation, there were significant differences between visual stimulation and olfactory–visual stimulation. Similar findings have also been found in previous studies on the effects of plant smellscapes on the human ANS. Jo et al. found that the naturally volatile odor of *Pinus densiflora* failed to have a significant impact on human blood pressure (BP) and pulse (P) values or on the values of other ANS indicators [52]. Fan et al. found that there were no significant differences in the variation in ANS-related indexes such as systolic blood pressure (SBP) and heart rate after the skin absorbed aroma substances of plants such as Rosa *setate* × *Rosa rugosa* [53].

In this study, only smelling Osmanthus had no significant effect on the autonomic nervous system, while simply viewing the Osmanthus landscape or viewing the landscape while smelling the Osmanthus odor led to significant increases in the SC index values of the autonomic nervous system. Previous research may be able to explain these results. Baer et al. found that odor stimuli did not cause significantly different cutaneous electrical responses [54]. This is consistent with the findings of this study, namely, that the SC values of the subjects after stimulation by the Osmanthus scent presented a downward trend, although this change was not significant. According to a study by Yuqian et al., human SC values increased significantly after subjects watched bamboo videos for 1–3 min [55]. This is consistent with the findings of the present study, namely that the short-term viewing of a garden landscape caused a significant increase in the subjects’ SC values, sympathetic nerve activity and emotional excitement. This finding also verifies the long-held belief that the visual system is the main method humans use to receive information from the external environment [56] and that it is also the most important sense organ in terms of yielding information about the outdoor environment [57].

In this study, the effect of visual stimulation on the BP, PP and P physiological index value of the autonomic nervous system was not significant, but it had a significant effect on the SC values. This phenomenon reminds us that when selecting physiological indicators, we need to consider their diversity to ensure the comprehensiveness of experimental results.

### 4.2. Effects of Olfactory, Visual and Olfactory–Visual Stimuli on the Central Nervous System

All three types of stimulation affected the central nervous system, which can be seen from the ANOVA results for the α and β brainwave amplitudes. The ANOVA results suggest that during exposure to the olfactory, visual and olfactory–visual stimulus modes, the subjects’ alpha and beta brainwave amplitudes exhibited significant increases compared with the control group. In addition, the visual stimulation group exhibited significantly higher increases in these amplitudes than the olfactory stimulation group, suggesting that compared with single-smell stimulation, the degree of influence of visual stimulation on the central nervous system was more obvious. This finding confirms the long-held belief that the visual system is the main method humans use to receive information from the external environment [56]. In addition, research in the field of neuroscience may also explain this phenomenon; olfactory neuronal transduction is the slowest among all the senses; olfactory detection takes about 400 milliseconds, which is ten times longer (i.e., slower) than visual detection [58,59]. Furthermore, the projection of olfactory images in the brain is significantly weaker than that of visual images. Specifically, there are about fifty thousand mitral cells for olfactory images, while there are one million pixels per visual image represented by retinal cells [60,61]. As a result, the human brain is relatively weak at processing olfactory cues in the absence of other cues [62].

Moreover, Fang M found that after subjects smelled the aroma of Osmanthus distillation extraction, their alpha wave amplitudes reduced significantly [63], a finding which does not agree with our study. The reasons for these differences are very complex and may be related to the source of the floral fragrance (from the natural release of flowers or distillation extraction), the concentration of the scent, the subjects’ sniffing threshold, sniffing duration and other factors [64]. This means that EEG technology is sensitive in sniff experiments, and the use of this technology may require more diverse test designs tailored to the characteristics of EEG activity.

### 4.3. Different Effects of Olfactory, Visual and Olfactory–Visual Stimuli on Human Physiological Indexes

In this study, it was found that the changes in the physiological indicators differed considerably among the different stimulation methods. These significant differences were recorded in both the autonomic nervous system (SC) and the central nervous system (α and β brainwaves).

From the perspective of SC, the SC values significantly increased under the olfactory–visual stimulus method, and this increase was significantly higher than that of the single olfactory stimulus method. It can be speculated that the olfactory–visual stimulus method caused a surge in the sympathetic nerve activity in the human ANS, which had a cumulative effect. This may mean that people become subconsciously (not subject to subjective consciousness) sympathetically excited when they smell and view garden plants, and this degree of excitement is much higher than when they purely smell or view these plants. Song found that forest-related olfactory–visual stimuli had cumulative effects on parasympathetic activity [13], a finding consistent with the results of this study to some extent.

In this study, in terms of CNS indicators and, in particular, the changes in α and β brainwaves caused by the Osmanthus fragrance, the olfactory–visual stimulus group recorded increases that fell between those of the olfactory and visual groups. Therefore, it can be concluded that the degree of activity of the human CNS when subjected to an olfactory–visual stimulus falls between that when subjected to a pure smell stimulus and a pure visual stimulus, a compromise effect. This indicates that the degree of the stability, relaxation and refreshing effects on the brain levels dominated by the subjective will when people smell Osmanthus and watch an Osmanthus landscape at the same time may fall between the degrees for pure sight and for pure smell. Research in the domain of neuroscience suggests that visual processing has a potential causal effect on olfactory perception [65]. The orbitofrontal cortex area in the brain is the site of a variety of sensory modalities, including olfactory and visual information [66,67,68]. Differential effects of individual sensory inputs can occur based on the interactions of these sensory modalities. In addition, in this study, the increases in α and β brainwave amplitudes under visual stimulation were significantly greater than those under olfactory stimulation. This is consistent with the conclusions of previous studies, which found that visual stimuli are the dominant sensory stimuli in a wide range of situations [69,70,71]. In general, people tend to believe that vision induces more stability, relaxation and refreshing effects than the other senses.

In this study, based on the above analysis, it can be determined that when the subjects were stimulated by Osmanthus-related olfactory–visual stimuli, they became very excited and subconsciously happy (ANS control), while such excitement was inhibited in their subjective consciousness (CNS control). As the ‘effect of images on olfaction’ is still poorly understood [25], the mechanism of this result is still unclear. In order to further explore this phenomenon, multidimensional sensory stimuli related to garden plant smells should be used in future research.

## 5. Conclusions

This study revealed that exposure to olfactory–visual stimuli of a garden plant odor and landscape relaxed and refreshed the subjects’ bodies to a certain extent, and this physiological health effect was greater with regards to the integrated response of the autonomic nervous system and central nervous system than the effect of only the visual or olfactory stimulus. Therefore, in the planning and designing of plant smellscapes in garden green space, it should be ensured that plant odors and corresponding landscapes are present at the same time in order to ensure the best health effect or, in other words, to ensure ‘It penetrates people’s hearts and spleens’.

Another finding of this study is that the CNS and ANS showed different physiological responses to the stimulus types associated with the smellscape. Under the olfactory–visual stimulation mode, the degree of the activity of the human central nervous system fell between that under the olfactory alone and only visual stimulation modes, with a compromise effect, while the degree of the excitation of the sympathetic nervous system was much higher than that under any of the single stimulus modes, with a cumulative effect being recorded.

All of these findings support the possibility that short-term exposure to olfactory–visual stimuli associated with garden plant smellscapes can induce physical and mental pleasure. From the late 1990s to the early 2000s, people’s interest in aromatherapy soared, but its influence is remains relatively limited. People need specific space and time and must pay certain costs to engage in aromatherapy. In today’s busy society, if people were to take in the interacting stimuli of the fragrant scent of garden plants and beautiful scenery when they visit park green spaces or walk in street green spaces, they may be able to relieve pressure and relax, could be very beneficial to public health. The results of this study provide valuable data for the realization of this possible approach, in addition to contributing to the development of a theoretical basis for the design of odor landscapes in garden plant landscapes.

However, this study is subject to some limitations. In terms of the study’s research objects, the subjects were mainly young college students; people of other age groups, education levels and living backgrounds were not involved. More population samples should be included in future studies to further validate the results of this study. Furthermore, although the study’s experimental design, which involved olfactory, visual and olfactory–visual stimuli, was scientific and innovative, there is still a variety of other components in garden green space environments, such as sound. Other environmental stimuli such as sound should be incorporated into future studies as evidence to support the health benefits of garden green space. In terms of the study’s measurement indicators, we only focused on the measurement of physiological indicators, not psychological indicators. Psychological indicators should be measured in future studies in order to verify the results of this study more comprehensively.

Although smell, which is directly related to aromatic odor, is the primary factor that triggers the perception of a smellscape, such perception not only involves the sense of smell but also the interaction of multidimensional senses. The future design of garden plant smellscapes should not only focus on odor elements but also make full use of all the perception elements related to smellscapes, i.e., those interacting multidimensional senses, so as to improve the environmental quality of garden green space and give full play to the health effects of garden plant smellscapes.

## Figures and Tables

**Table 1:** Changes in physiological indexes before and during olfactory, visual and olfactory–visual stimulation.

Stimulus Type	Variable (Unit)	Before		During		T
		Mean	SD	Mean	SD	
**O**	SBP (mm Hg)	108.06	10.91	107.88	11.14	0.185
**V**		105.89	11.14	104.91	9.81	0.603
**O&V**		105.80	7.92	105.22	7.45	0.579
**C**		106.54	9.15	104.94	7.10	1.406
**O**	DBP (mm Hg)	62.88	8.41	67.25	10.10	3.400 **
**V**		63.48	6.70	63.89	9.32	−0.203
**O&V**		61.04	6.93	64.30	6.48	−2.783 **
**C**		61.00	7.93	62.46	5.85	−1.421
**O**	PP (mm Hg)	45.19	8.29	40.63	7.05	3.518 **
**V**		42.41	8.58	41.02	10.34	0.577
**O&V**		44.76	4.84	40.92	5.17	3.373 **
**C**		45.54	6.84	42.48	5.15	2.899 **
**O** **V** **O&V**	P (bpm)	77.67	12.56	75.33	11.40	2.874 **
		73.96	11.82	73.80	11.29	0.160
		72.28	9.95	71.56	10.19	0.946
**C**	SC (µΩ)	74.38	10.40	74.40	7.86	−0.21
**O**		2.94	2.92	2.78	2.68	0.969
**V**		3.03	2.17	3.22	2.18	−1.774
**O&V**		2.95	2.14	3.40	2.48	2.824 **
**C**		3.35	2.05	2.88	2.06	3.284 *
**O**	α waves (µV)	17.67	6.64	18.04	4.55	−0.303
**V**		13.90	7.19	20.10	9.45	−4.785 **
**O&V**		16.03	5.03	18.31	6.77	−2.241 *
**C**		19.78	17.16	14.60	10.52	2.486 *
**O**	β waves (µV)	11.49	4.47	11.83	5.48	−0.374
**V**		8.27	5.09	13.78	6.79	−6.067 **
**O&V**		10.12	4.35	11.52	4.87	−2.153 **
**C**		13.37	12.65	10.11	9.15	2.537 *

*p* < 0.01 indicates an extremely significant difference; 0.01 < *p* < 0.05 indicates a significant difference; *p* > 0.05 indicates that the difference was not significant. O: olfactory stimulation; V: visual stimulation; O&V: olfactory–visual stimulation; C: control; SBP: systolic blood pressure; DBP: diastolic blood pressure; PP: pulse pressure difference; P: pulse; SC: skin conductance. ** indicates that the difference between the two was extremely significant, *p* < 0.01.

**Table 2:** Significance analysis of the influence of different stimulation methods on skin conductance, α waves and β waves (SC, α waves and β waves).

		P (LSD)		
		O	V	O&V
SC	O&V	0.004	--	--
	C	--	0.001	0.000
αwaves	O	--	0.006	--
	C	0.009	0.000	0.001
βwaves	V	0.001	--	0.006
	C	0.010	0.000	0.001

*p* < 0.01 indicates an extremely significant difference; 0.01 < *p* < 0.05 indicates a significant difference; *p* > 0.05 indicates that the difference was not significant. Those not shown were a combination of *p* > 0.05. O: olfactory stimulation; V: visual stimulation; O&V: olfactory–visual stimulation; C: control; SC: skin conductance.

## Data Availability

Data available on request from the authors.
